# Obscure Symptoms of Syphilis and So-Called Parasyphilitic Diseases, with Discussion of Treatment

**Published:** 1917-02

**Authors:** J. T. M’Glasson

**Affiliations:** Waco, Texas


### Obscure Symptoms of Syphilis and So-Called Parasyphilitic Diseases, With Discussion of Treatment.

BY J. T. M'GLASSON, M. D., WACO, TEXAS.

At the outset, I wish to state that I do not believe the term, parasyphilitic disease, should have any place in our nomenclature. A certain condition is syphilitic, or it is not. There can be no middle ground. The term here is only used in the title to make my meaning clear to those who may hold the other conception. My position in this matter is very generally upheld by a majority of syphilographers at this time. The reason for this is well set forth by Barker, by the demonstration of treponema pallidum in all kinds of syphilitic lesions, including not only the chancre of primary syphilis and the gummata of tertiary, but also in the walls of aortic aneurysms (Schmirl, J. Homer Wright), in the brain substance in general paresis (Noguchi and Moore), in lesions of tabes dorsalis (Morinesco), and in the organs of children dead of congenital syphilis (including germs of developing teeth), and in placentas of the mothers of syphilitic children by various observers.

I also want to say, I will not trouble you with common symptoms of a typical case of syphilis, but I want to stress symptoms that are very suggestive of syphilitic infection which are not commonly given their proper value, that we may be on our guard. No other disease so generally invades all fields of medicine as does syphilis. The syphilographer is therefore dependent on every branch of medicine in diagnosis. If, therefore, I may lay too much stress on the eye men, in this paper, it is because of their peculiar opportunities of getting luetics who may otherwise escape observation until too late. My object is in the line of preventive medicine. I want to call the attention of the profession to what may be termed the "preneurotic" symptoms. In attempting this, I am not unmindful of the magnitude of the subject, or the difficulties confronting us in determining what may be called "preneurotic" symptoms.

Kolmer, in the *Journal of the American Medical Association* of May 6, 1916, has said: "In view of the recent researches in syphilis disclosing the spirocheta pallida in the tissues in tertiary lesions, it is very doubtful whether it is permissible to speak of the sequelae of syphilis. The toxins and other products of the spirochetes may produce degenerative changes in various organs, but it is more likely that the late consequences of a syphilitic infection, as aneurysm, aortic insufficiency, arteries, etc., may be direct lesions of syphilis, due to the presence of living spirochetes rather than sequelae in the true meaning of the term." Noguchi and Moore, Nickols, Wile and others have demonstrated the microparasites in the brain and spinal cord of paresis and tabes dorsalis, thereby rendering obsolete the terms "parasyphilitic" and "pseudo-syphilitic," and Warthin and others have likewise shown the parasites in the heart and vessel walls even in those who during life were regarded as cured. It is probable that there are different strains of the spirocheta pallida with different tissue affinities; thus a certain strain may have a selective infinity for the tissues of the central nervous system, and its host show the results of early or precocious involvement of these tissues; another strain may have a selective affinity for the cardiovascular system; another for the bones and periosteum or the skin and mucous membranes, etc., the seriousness of an infection depending on the question of strain infection. Thus the older syphilographers taught that persons showing well developed cutaneous lesions were less likely to develop lesions in the internal organs and were more amenable to treatment. The question then arises, are there not certain symptoms suggestice of preneurotic syphilis? —symptoms even diagnostic of beginning syphilitic neuroses.

Our present state of knowledge does not admit of a definite answer to this question. but we will all agree that the imperfectly treated syphilitic and those who have had no treatment are liable at some time in life to come down with some of the syphilitic neuroses. We also know that a majority of our tabetics do not know that they are syphilitic and will combat the physician, attempt to make out such a history, at times taking umbrage, at the mere suggestion. That they have had little or no treatment goes without saying. Other cases will give a good history of clinical symptoms, with a short or irregular treatment; while a certain number will come down with syphilitic neuroses, and die, in spite of the most heroic treatment.

With the above statements before us, it is pertinent to ask, Where are we at? Are there not some symptoms which will enable us to recognize that not only these patients are syphilitic, but that they are cases of probable beginning or "preneurotic"

syphilis? Of course, it is not possible to say that, any given case will go on to tabes, but we should, in the light of our present knowledge, proceed on that assumption.

With the above limitations, I shall, in this paper, attempt to answer, in the affirmative: First, I want to call your attention to some conclusions reached by Barker: (a) Of the extent of unsuspected syphilis in the community; (b) of the fact that the majority of cases treated, even those believed to be thoroughly treated, are not cured; (c) that many of the wives and children of supposedly cured luetics are infected, even though they show no symptoms; (d) that the reason why the syphilitic child of a syphilitic father can be nursed by its mother without giving the disease to the mother (Colles law) is that the mother is already infected, the child having been infected by placental transmission from the mother rather than by direct inheritance from the father; and (e) that the reason why the apparently healthy child of luetic parents seems to be immune from infection, say, from an infected wet nurse (Profetas law), lies in the fact that the child is already infected. Another statement I want to make is, that it is now proven that the treponema becomes widely distributed throughout the body before the initial lesion develops. In other words, that, by the time a Hunterian chancre appears, the syphilis has, as a rule, become generalized, and the excision of the chancre, or any other form of abortive treatment, is of no value.

Before going further, I want to say that only since the widespread use of the sero reactions, in the diagnosis of syphilis, have we become mindful of the extent of syphilis, and also, since its introduction, have we been able to tell with any degree of certainty when our patients were cured. But I want to take issue with those who are wont to rely implicity on the Wassermann. Wolbarst and others have shown that it is dangerous to accept a positive Wassermann without other confirmation. Osler's dictum: "That laboratory methods (Wassermann) are an addition to, and not a substitute for, clinical observations" is as true in serology as in other work. Simply accept it for what it is worth. A positive Wassermann can be accepted with greater confidence than a negative one, bearing in mind the conditions that give a positive Wassermann, other than syphilis. Keyes has gone so far as to say a positive Wassermann, in the absence of clinical symptoms, and history, would not be a bar to marriage.

In preparing my paper, I made out the following questions and sent them to thirty-five eye men. A summary, together with the questions, would be as follows. Facts are gathered from twenty-one answers that were satisfactory for this report.

Question 1.—What per cent of cases of optic neuritis and choked disc do you find are syphilitic?

Answer.—An average of 63 per cent. One man answered as low as 10 per cent. Eight answered above 70 per cent. I believe that this is a very low average, and suggest that the oculist should follow these cases up. I am certain they will find cases of syphilis where they are least expected.

Question 2.—What per cent of cases come to you without other suggestive symptoms of syphilis?

Answer.—Sixty-two per cent. Note the vast number of which this is the only symptom.

Question 3.—What per cent of such cases are hereditary?

Answer.—Twenty-seven per cent. I think this sufficiently low.

Question 4.—At what age do you find eye changes most frequently?

Answer.—Ten to thirty years by 7; 20 to 40 by 6; 40 to 70 by 4; 30 to 50 by 1; any age by 2. Thirteen found a majority under 40. Five found over that age; while two answered any age. My own experience is under 40.

Question 5.—Are there any other eye changes that, to your mind, suggest syphilis? This is only intended for first symptoms; not for conditions where syphilis is already in evidence.

Answer.—Fundus changes, iritis, paralysis of extra and intraocular muscles, keratitis, inflammations of uveal tract, retinochoroiditis, strabismus, anisocoria, excessively premaute failrue of accommodation, etc.

My attention was called to this work by six cases referred to me by oculists, all of whom denied any knowledge of syphilis. Their parents also vehemently denied it, but subsequent results demonstrated the accuracy of our findings. A case in point.

Case 1.—Girl, age 14, referred by Dr. J. R. Ferrell of our city, who reported that she had edema of retina or choroid retinitis, with vision 32/100 right, 20/80 in left eye. After considering the case, and after diligent questioning, found definite history of syphilis. Luetic treatment resulted in much relief, vision more than doubled, patient returning to school, continuing full course of studies. Treatment continued.

Another case of similar nature, but showing other side of picture.

CASE 2.—Boy, age 12, referred by Dr. L. F. Naylor of our city. Eye findings were widely dilated pupils; no response to light. Choked discs; only had light perception. Ophthalmoscopic examination difficult, because of extreme nausea and vomiting from reflected light. Wassermann positive. Patient's father refused to accept our diagnosis, and refused treatment. Patient died one month later in convulsions. I do not believe that this patient could have been helped at this late date, but at the beginning of the trouble, if properly diagnosed, I believe we would have had another story to tell.

Another case in which the diagnosis of locomotor ataxia was borne out by time.

CASE 3.—J. S., age 47, was seen by Dr. Jno. L. Burgess of this city two or more years ago. He had at that time paralysis of external rectus, possibly involving superior oblique, and some irritation of optic nerve. He was told that it was syphilitic and advised to take proper treatment at once. He failed to do so. I was consulted this spring, when it was found he was tabetic, advanced, and I can give him very little encouragement. I believe a different picture would be presented had he taken the proper treatment.

It is proper to ask now, what other conditions should suggest luetic involvement? I can only answer, that there are no parts of the anatomy exempt. But there are conditions which are more likely to be syphilitic than others; i. e. (1) aneurysms, (2) certain grave anemias resembling the Addison Riermier type, (3) strictures of rectum; (4) thickening of or nodules in the bones, especially when accompanied by night pains; (5) repeated miscarriages without explainable cause; (6) and obscure nervous cases, especially where the symptoms come and go; (7) disorders of metabolism and of internal secretions (Barker); (8) stomach crises with obscure pain, mild or severe, with or without vomiting.

CASE 4.—Female, age 21, fairly well nourished; had stomach crises, occurring once or twice monthly, at times so severe as to be much prostrated for several days. The attacks came on following the simplest diet, or even with a milk diet. While she never at any time vomited blood, still the condition was suggestive of ulcer or malignancy. She was X-rayed by Dr. R. B. Nutter of our city, with the following findings. I am indebted to Dr. Nutter, who very materially assisted me in getting this data: An abnormal thoracic aorta shadow with the Roentgenoscope. This sign was first described by Keinback, of Vienna, and serum tests have proven its value. The shadow is a moderate increase in density, with an increase in breadth up to 30 per cent. Stomach after barium meal showed to be moderately dilated, hookshaped, marked ptosis, hypomotility, outlines regular, pylorus freely movable, no spasm nor filling defects, rapid emptying without definite cycles. This, in spite of decided hypomotility. This is due to an open pylorus. This picture may also be seen in lead poisoning and achylia gastrica. This patient under antiluetic treatment was markedly improved, and has not had a gastric crises since.

Case 5.—Male, age 42; very similar to case 4. Periodical stomach crises, with rather constant pain over pylorus, nausea and vomiting, easily induced, not explainable by character of food; no blood in vomitus. This case was very suggestive of stomach ulcer. X-ray findings by Dr. R. B. Nutter were the same as case 4, in regard to aorta; so much so that I advised looking for it in all suspicious cases. Stomach, after barium meal, showed to be hookshaped, normal position, regular outlines, no spasm, no filling defects, pylorus movable, peristalsis faint, definite hypomotility, in spite of which there was rapid emptying. Both of these cases presented the same definite hypomotility, deficient peristalsis, yet emptying in half the time of a normal stomach.

Dr. Barker, of Baltimore, reports case of man, age 34 years, who was much emaciated, neurasthenic and with hypomaniacal symptoms; had facial tic; single epileptiform convulsions, and insomnia, with a positive Von Graefe and Rosenbach sign. Under observations in hospital, the patient was euphoric, mentally restless, and overalert, with a tendency to hurried speech, talkativeness, and general pressure of activity. This patient had, while in hospital, a generalized epileptiform convulsion, ushered in by a loud cry. The lips and tongue were bitten and the face cyanotic. There was no incontinence of urine or feces. The convulsive seizure lasted two minutes and was followed by coma, lasting for some five minutes, after which patient awoke and was temporarily disoriented as to time and place, and had no memory of the seizure. Under salvarsan, followed by salicylate of mercury, patient rapidly gained in weight, felt well and had no more convulsions, so that obscure epileptiform seizures should be classed among those suggesting syphilis.

Case 6.—L. J. L., male, age 34. Patient first seen by me in May of this year, with the following history: The initial lesion four years ago was given four doses of salvarsan and pronounced cured. During last year had double vision, with trouble in moving around in the dark, as he expressed it. Some difficulty in negotiating stairways. In the early part of this year had a gradually increasing incontinence of urine until he lost control entirely. He was in this condition when I saw him in May, 1916. His condition then was a very marked Babinski; would fall if not held up. Patella reflexes abolished, marked ankle clonus. Vision defects marked. Eye grounds showed choked discs, atrophy of the optic nerve; had three eye men see him. The first thing patient remembers of anything disturbing him was his double vision. This was more than a year before I saw him, and it goes without saying his treatment would have been much simplified had we seen him then. His whole aspect was a typical tabetic. This patient has gained thirty-seven pounds in weight, and has no vision defects noticeable. Shoots rifle and shotgun perfectly and runs his own auto, and his Babinski is negligible. I should have said that he had a four plus blood Wassermann and one plus spinal fluid, a low globulin increase and three cells per field. This undoubtedly accounts for his rapid improvement. No incontinence at this time, and patient working every day. Treatment was mixed salvarsan and intravenous mercury.

Barrett, of Ann Arbor, has recently reported a number of cases of manic depressive insanity, and attempts to prove the luetic etiology of these conditions. I do not believe his point well taken. I think the luetic conditions were simply coincident. As proof, I submit that they should be cured or improved by anti-syphilitic treatment, whereas such is not the case. Treatment being continued until a negative Wassermann was had, conditions mentally just the same, simply having records of improvement followed by exacerbation.

In treatment, I want to suggest that we must recognize that the arsenicals will not cure syphilis. That they are one of my most potent weapons in controlling symptoms by their ability to inhibit the activity of the treponema pallidum is recognized, but their therapeutic effect stops here. Laredde, of Paris, carried patients through series of salvarsan treatment consisting of from thirty to fifty doses, but found the Wassermann after periods of negative reactions would return positive. Dr. C. P. Schenck of our city referred me a case of pharyngeal ulcer luetic in a patient who had twelve doses of salvarsan. Last dose one year preceding his visit to me. So we must give mercury as if the arsenicals had not been given. I prefer hypodermic medication. In fact, I have gotten results with the soluble mercury, bichloride, that compares favorably with the newer arsenicals. In giving bichloride it must be given in large doses, one-third of a grain every day. For the continuous treatment, salicylate of mercury in one-grain doses twice weekly gives me the most satisfactory treatment, although some patients do well on inunctions. Each case will be more or less a law unto itself.

Don't be guilty of giving your patients four or five doses of salvarsan and tell them they are cured of their syphilis, for they are sure to make your life unhappy. Sometimes because of pain it is difficult to get patients to take the bichloride, but they should not expect to pay the penalty of indiscretions on flowery beds of ease.

